# PTX3 Effects on Osteogenic Differentiation in Osteoporosis: An In Vitro Study

**DOI:** 10.3390/ijms22115944

**Published:** 2021-05-31

**Authors:** Chiara Greggi, Ida Cariati, Federica Onorato, Riccardo Iundusi, Manuel Scimeca, Umberto Tarantino

**Affiliations:** 1 Ph.D. in Medical-Surgical Biotechnologies and Translational Medicine, “Tor Vergata” University of Rome, via Montpellier 1, 00133 Rome, Italy; chiara.greggi@gmail.com (C.G.); ida.cariati@uniroma2.it (I.C.); 2 Department of Clinical Sciences and Translational Medicine, “Tor Vergata” University of Rome, via Montpellier 1, 00133 Rome, Italy; 3 Department of Orthopaedics and Traumatology, “Policlinico Tor Vergata” Foundation, viale Oxford 81, 00133 Rome, Italy; fede.onorato@gmail.com (F.O.); riccardo.iundusi@uniroma2.it (R.I.); 4 Department of Biomedicine and Prevention, “Tor Vergata” University of Rome, via Montpellier 1, 00133 Rome, Italy; manuel.scimeca@uniroma2.it

**Keywords:** PTX3, osteoporosis, osteoblasts, osteogenic differentiation, mineralization, calcification

## Abstract

Pentraxin 3 (PTX3) is a glycoprotein belonging to the humoral arm of innate immunity that participates in the body’s defence mechanisms against infectious diseases. It has recently been defined as a multifunctional protein, given its involvement in numerous physiological and pathological processes, as well as in the pathogenesis of age-related diseases such as osteoporosis. Based on this evidence, the aim of our study was to investigate the possible role of PTX3 in both the osteoblastic differentiation and calcification process: to this end, primary osteoblast cultures from control and osteoporotic patients were incubated with human recombinant PTX3 (hrPTX3) for 72 h. Standard osteinduction treatment, consisting of β-glycerophosphate, dexamethasone and ascorbic acid, was used as control. Our results showed that treatment with hrPTX3, as well as with the osteogenic cocktail, induced cell differentiation towards the osteoblastic lineage. We also observed that the treatment not only promoted an increase in cell proliferation, but also the formation of calcification-like structures, especially in primary cultures from osteoporotic patients. In conclusion, the results reported here suggest the involvement of PTX3 in osteogenic differentiation, highlighting its osteoinductive capacity, like the standard osteoinduction treatment. Therefore, this study opens new and exciting perspectives about the possible role of PTX3 as biomarker and therapeutic agent for osteoporosis.

## 1. Introduction

In the early 1990s, pentraxin 3 (PTX3), the prototype of long pentraxin, was first identified. It is a soluble pattern recognition molecule (PRM), released by myeloid lineage cells (dendritic cells, macrophages and monocytes) and stromal cells (epithelial cells, smooth muscle cells, chondrocytes and fibroblasts) in response to primary pro-inflammatory cytokines, damage-associated molecular patterns (DAMPs), agonists of Toll-like receptors (TLRs), microbial components or intact microorganisms [1,2,3,4,5].

PTX3 is a molecule known for its involvement in modulating the mechanisms of innate immunity, which come into play to protect the body from infectious diseases [6,7,8]. Not surprisingly, numerous studies have shown over the years that Ptx3-deficient mice are more susceptible to viral, bacterial and fungal infections [9,10,11]. PTX3 is also considered a multifunctional glycoprotein involved in several biological processes, both physiological and pathological [1]. Indeed, in vivo and in vitro studies have shown that PTX3 binds and sequesters fibroblast growth factor 2 (FGF2) at its N-terminal end, thereby inhibiting FGF2-dependent proliferation of endothelial and smooth muscle cells during the neo-angiogenesis process [12,13]. This inhibitory effect is reversed by the binding of PTX3 to tumour necrosis factor-stimulated gene-6 (TSG-6): preventing the interaction between PTX3 and FGF2, TSG-6 plays a pro-angiogenic function and can therefore be considered as a new modulator of neovascularization [14]. Moreover, in several mouse models of tissue damage, PTX3 has been shown to play a key role in the damage response by promoting pericellular fibrinolysis, interacting with fibrin and plasminogen, and allowing tissue repair [15]. In 2018, Bonfiglio and colleagues suggested PTX3 as a new potential marker of poorly differentiated breast carcinomas, having found significantly higher expression of this molecule in the environment of malignant lesions characterised by microcalcifications, compared to malignant lesions without calcifications [16,17]. Finally, a possible role of PTX3 in bone metabolism has also been suggested, although few and conflicting data are currently available. In this regard, we have recently investigated the possible role played by PTX3 in bone homeostasis, comparing the expression and function of this molecule in human osteoblasts from osteoporotic, osteoarthritic and control patients [2]. Our results showed that there is a close association between bone health and the number of osteoblasts expressing PTX3. Noteworthy, differential expression of PTX3 in the bone tissue of the three experimental groups was shown, with significantly lower PTX3 levels only in osteoporotic patients, whose osteoblasts were less likely to generate hydroxyapatite crystals. Furthermore, we have shown that treatment with anti-PTX3 antibody significantly affects the behavior of osteoblasts, which lose the morphological and molecular characteristics of mature osteoblasts, acquiring a fibroblast-like phenotype and significantly reducing receptor activator of nuclear factor kappa-B ligand (RANKL) and Runt-Related Transcription Factor 2 (RUNX2) expression. Overall, our results suggested the involvement of PTX3 in osteoblast proliferation, differentiation, and function, thus confirming its central role in bone homeostasis [2]. Visconti and colleagues give further support to these results, as their study showed that healthy subjects and patients suffering from osteoarthritis and osteoporosis, are characterised by different PTX3 serum levels, again suggesting the involvement of this molecule in bone metabolism and how it may represent a new potential marker for bone-related phenotypes [18]. Further evidence that PTX3 is a molecule involved in the osteogenic differentiation process comes from Kim and colleagues, who demonstrated that knockdown of PTX3 impairs human dental pulp stem cells differentiation towards the osteogenic/odontogenic lineage, thereby compromising the regeneration and repair processes mediated by these cells [19]. On the other hand, Liu and colleagues studied the effect of PTX3 overexpression by the lentiviral system in MC3T3-E1 cells, finding a significant increase in the expression of osteoblasts signature genes such as RUNX2, alkaline phosphatase (ALP), osteocalcin (OCN) and osterix (OSX); there results further highlight the role of this molecule in osteoblastic differentiation process [20].

As the average age of the population increases, the identification of new potential diagnostic markers or therapeutic targets for age-related diseases is becoming increasingly important. To this end, the main aim of this study was to investigate the osteogenic role of PTX3 by evaluating the effect of human recombinant PTX3 (hrPTX3) in primary cultures of human osteoblasts derived from osteoporotic (OP) and control (CTRL) patients. This experimental design will allow to both better elucidate the involvement of PTX3 in the bone mineralization process and demonstrate how PTX3 may represent a valid future therapeutic choice for the treatment of osteoporosis.

## 2. Results

### 2.1. Histomorphometrical Analysis

**Figure 1:**
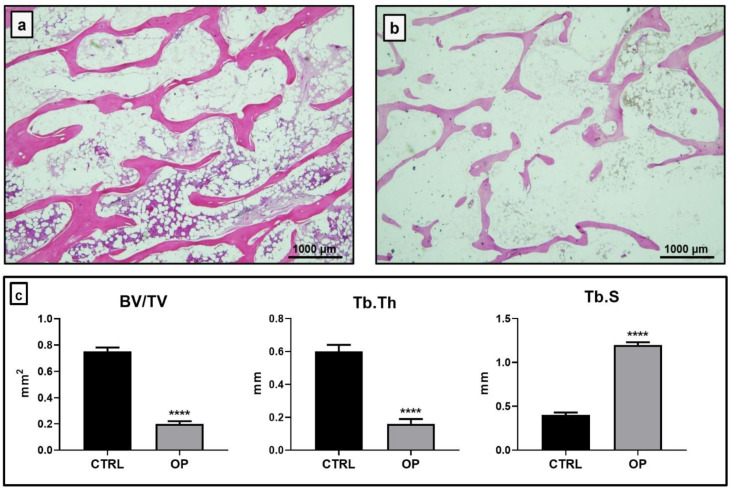
Haematoxylin and eosin (H&E) sections of bone head biopsies and evaluation of bone morphometric parameters. (**a**) Histomorphometrical analysis showed a conserved trabecular network in control (CTRL) patients. (**b**) Osteoporotic (OP) patients displayed a remarkable loss of both trabecular thickness and trabecular number. (**c**) The analysis of bone quality parameters evaluated by NIS-Elements software showed a significant reduction of bone volume (BV/TV) and trabecular thickness (Tb.Th) in the OP group compared to the CTRL group (**** *p* < 0.0001). In contrast, Tb.S values were significantly reduced in CTRL patients compared to OP patients (**** *p* < 0.0001). (H&E 2x, scale bar represents 1000 μm).

The analysis of conventional bone morphometric parameters, such as bone volume (BV/TV), trabecular thickness (Tb.Th) and trabecular separations (Tb.S) was carried out through the use of NIS-Elements software in haematoxylin and eosin (H&E) sections (Figure 1a,b). The histomorphometric analysis showed a significant difference between the two groups of patients for each parameter considered (**** *p* < 0.0001) (Figure 1c). Specifically, BV/TV was significantly increased in the CTRL group (0.75 ± 0.03 mm^2^) compared to the OP group (0.21 ± 0.02 mm^2^). The same result was also obtained for the Tb.Th parameter, with mean values of 0.61 ± 0.04 mm for CTRL patients and 0.16 ± 0.03 mm for OP patients. On the contrary, we measured significantly reduced Tb.S values for the CTRL group (0.41 ± 0.03 mm) compared to the OP group (1.22 ± 0.03 mm).

### 2.2. Immunohistochemical Analysis

**Figure 2:**
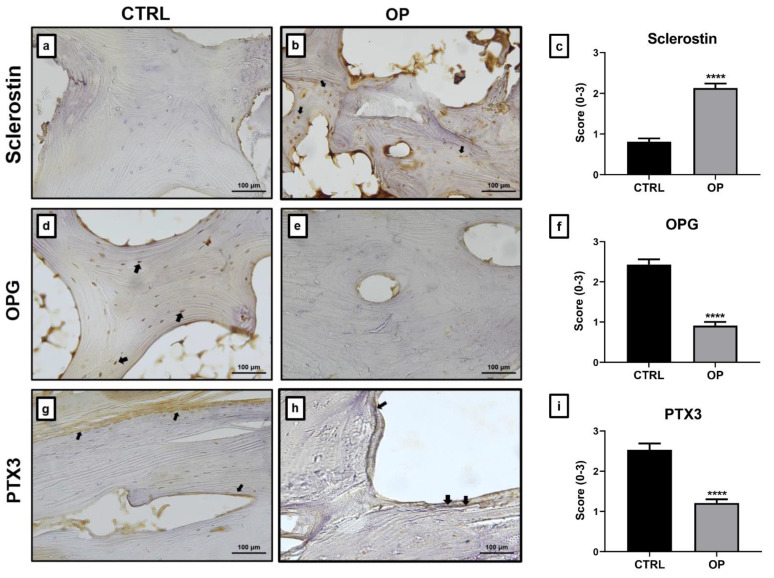
Sclerostin, osteoprotegerin (OPG) and pentraxin 3 (PTX3) expression. Immunohistochemical analysis of sclerostin, OPG and PTX3 expression in bone head biopsies. (**a**) Image displays few sclerostin-positive osteocyte cells in CTRL patients, (**b**) while OP patients showed numerous sclerostin-positive osteocytes (arrows). (**c**) Graph shows immunohistochemical results for sclerostin expression (CTRL vs. OP, **** *p* < 0.0001). (**d**) Numerous OPG positive osteoblast were present in the trabecular bone of CTRL patients (arrows), (**e**) while OP patients showed low OPG expression. (**f**) Graph shows immunohistochemical results for OPG expression (CTRL vs. OP, **** *p* < 0.0001). (**g**) CTRL patients showed PTX3 expression both in osteoblasts and bone marrow cells (arrows), (**h**) while OP bone tissue showed significantly lower PTX3-positive osteoblasts. (**i**) Graph displays immunohistochemical results for PTX3 expression (CTRL vs. OP, **** *p* < 0.0001). 20x images, scale bar represents 100 μm.

The immunohistochemical analysis of the bone tissue was performed using a semiquantitative method that assign a score from 0 to 3 according to the number of positive osteocytes for sclerostin and osteoblasts for osteoprotegerin (OPG) and PTX3. Figure 2 shows statistically significant differences between the two experimental groups (**** *p* < 0.0001). In fact, the relative amount of sclerostin-positive cells was significantly higher in OP patients (2.13 ± 0.11) than in CTRL patients (0.81 ± 0.08) (Figure 2a–c); while the number of osteoblasts expressing OPG was significantly lower in the OP group (0.91 ± 0.09) than in the CTRL group (2.43 ± 0.13) (Figure 2d–f). Confirming the data previously obtained [2], immunohistochemical analysis of femoral head biopsies showed a differential expression of PTX3 in the two experimental groups (**** *p* < 0.0001). Specifically, we found a significantly lower relative number of PTX3-positive osteoblasts in the bone tissue of OP patients (1.21 ± 0.09) compared to CTRL patients (2.53 ± 0.16) (Figure 2g–i).

### 2.3. Human Recombinant Pentraxin 3 (hrPTX3) and Osteogenic Cocktail Treatment on Primary Osteoblasts Cultures

**Figure 3:**
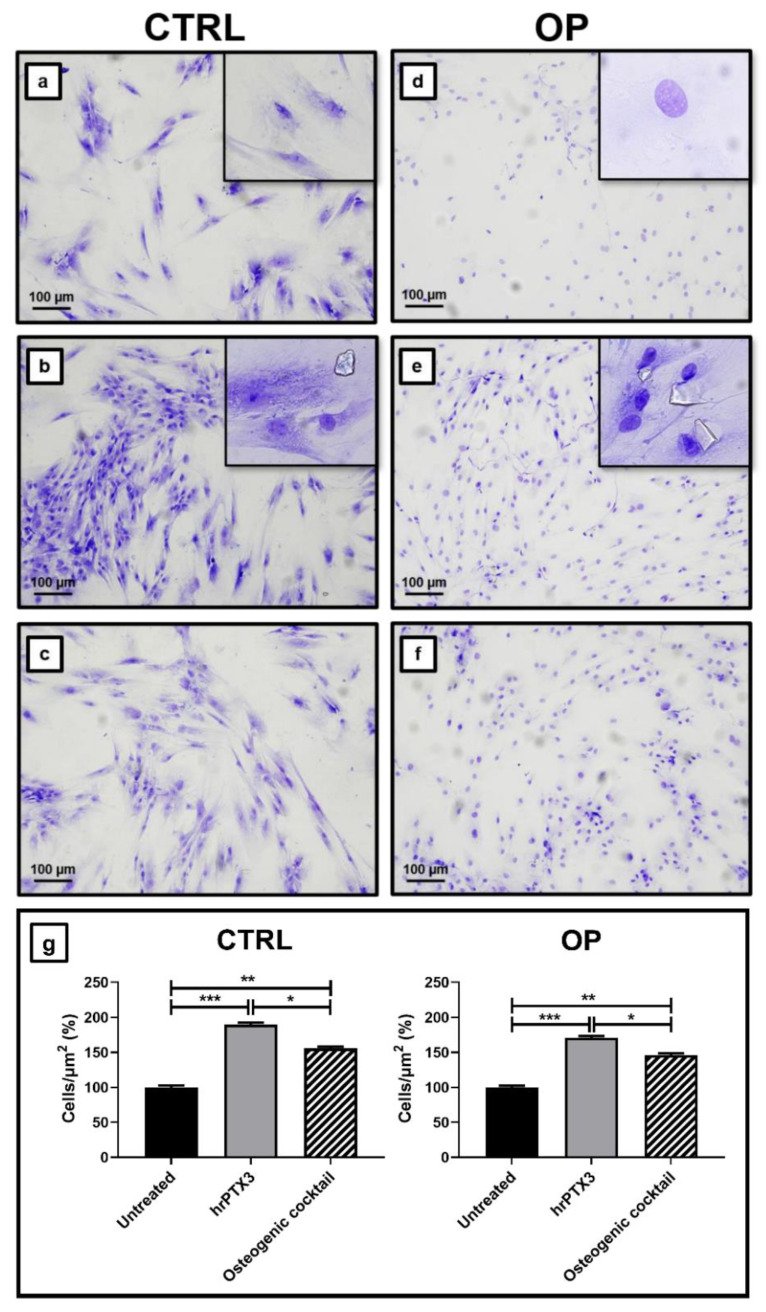
Effects of hrPTX3 and osteogenic cocktail treatment on primary osteoblasts cultures. The effects of hrPTX3 and osteogenic cocktail treatment on cell differentiation and proliferation were assessed by Toluidine blue staining. (**a**) Untreated osteoblasts isolated from CTRL patients showed the classic phenotype of a mature osteoblast. (**b**) Treatment with hrPTX3 induced the formation of cell clusters and calcification-like structures. (**c**) The inductive effect on proliferation and clustering was also evident following treatment with osteogenic cocktail. (**d**) Untreated osteoblasts isolated from OP patients showed a fibroblast-like phenotype. (**e**) Treatment with hrPTX3 promoted differentiation towards the osteoblastic lineage and formation of numerous calcification-like structures. (**f**) The osteogenic cocktail also had an inductive effect on proliferation. (**g**) Graphs show the effect of treatments on proliferation in both CTRL and OP patient cultures. 10x images, scale bar represents 100 μm (*** *p* < 0.001, ** *p* < 0.01, * *p* < 0.05).

Toluidine blue staining allowed us to evaluate important morphological differences between the two groups of patients under the three experimental conditions. Regarding the untreated cultures, we noted that the osteoblasts isolated from the bone tissue of CTRL patients were characterised by a classic morphology like that of a mature osteoblast (Figure 3a), while the osteoblasts of primary cultures derived from OP patients showed a reduced nucleus/cytoplasm ratio with a similar fibroblast appearance compatible with an undifferentiated pre-osteoblastic cell (Figure 3d). Surprisingly, after 72 h of treatment with hrPTX3, OP primary cells acquired a phenotype consistent with a mature osteoblast (Figure 3e), a less obvious effect in cultures of CTRL patients who showed this phenotype already in the untreated condition (Figure 3b). Noteworthily, this effect is the same as that obtained in cultures treated with osteogenic cocktail, both in CTRL and OP groups (Figure 3c,f), showing that PTX3 possesses considerable osteoinductive capabilities. We also observed that treatment with hrPTX3 resulted in a significant increase in proliferation, both compared to the untreated condition and to cultures treated with the osteogenic cocktail, in both CTRL and OP cultures (Figure 3g). Indeed, osteoblasts from CTRL patients treated with hrPTX3 and the osteogenic cocktail showed an increase in the proliferation rate expressed in cells/μm^2^ (%) of 90% and 56%, respectively, compared to the untreated condition (untreated vs. hrPTX3, *** *p* < 0.001; untreated vs. osteogenic cocktail, ** *p* < 0.01; hrPTX3 vs. osteogenic cocktail, * *p* < 0.05). Similarly, we observed for OP patient cultures an increase in proliferation of 71% after treatment with hrPTX3 and 46% after treatment with osteogenic cocktail, compared to untreated cells (untreated vs. hrPTX3, *** *p* < 0.001; untreated vs. osteogenic cocktail, ** *p* < 0.01; hrPTX3 vs. osteogenic cocktail, * *p* < 0.05).

In the two experimental groups, the increase in proliferation seems to accompany the acquisition by both CTRL and OP osteoblasts of the ability to organize into cell clusters (Figure 3). Finally, Toluidine blue staining allowed us to see that treatment with hrPTX3 influenced the ability of osteoblasts to form small calcification-like structures. This effect was significant in cultures of osteoblasts isolated from OP patients, compared to CTRL patients, where such structures were found to be present in all experimental conditions.

**Figure 4:**
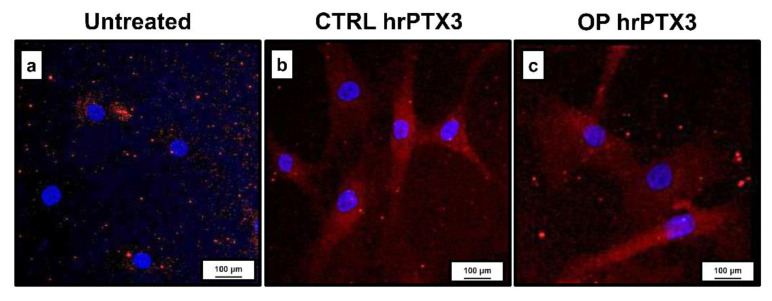
Immunostaining for PTX3 on CTRL and OP primary osteoblasts culture. (**a**) In untreated cultures, a slight expression of PTX3 can be observed in a few cells. In both CTRL (**b**) and OP (**c**) treated cultures, the uptake of hrPTX3 is observed at the cytoplasmic level. Small autofluorescent calcification-like structures are also observed. 40x images, scale bar represents 100 μm.

The uptake of hrPTX3 in CTRL and OP cultures is shown in Figure 4.

### 2.4. Effect of Treatment with hrPTX3 and Osteogenic Cocktail on the Calcification Process

**Figure 5:**
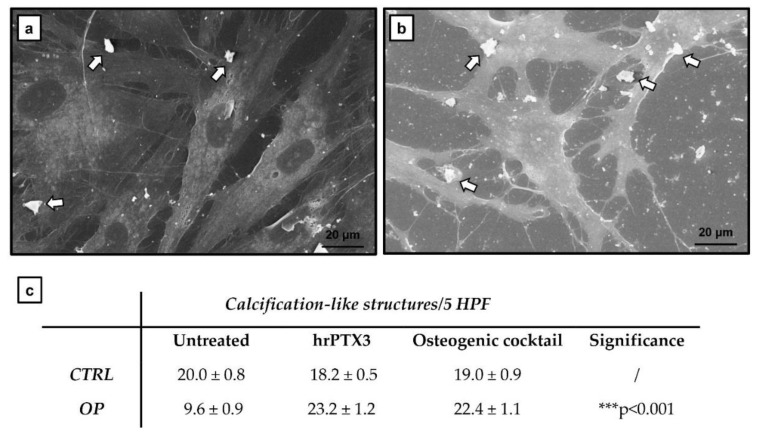
Scanning electron microscopy (SEM)–energy-dispersive X-ray spectroscopy (EDX) microanalysis on human osteoblast primary cultures. (**a**,**b**) Representative image of CTRL and OP cultures treated with hrPTX3 and osteogenic cocktail; calcification-like structures (arrow) can be seen. Scale bar 20 μm. (**c**) Evaluation of calcification-like structures amount.

Since several studies in the literature reported that PTX3 is a molecule involved in the mineralisation process, and given our results obtained previously [2], we evaluated the effect of treatment with hrPTX3 and osteogenic cocktail on primary osteoblast cultures by scanning electron microscopy–energy-dispersive X-ray spectroscopy (SEM-EDX) microanalysis. This analysis not only confirmed that the cultures treated with hrPTX3 and osteogenic cocktail were characterised by well-differentiated osteoblasts, but also allowed us to observe the presence of calcification-like structures, both isolated from CTRL (Figure 5a) and OP patients (Figure 5b). Furthermore, it was found that, after 72 h of treatment, the number of such structures was higher in cultures isolated from OP patients than in CTRL patients, both in the experimental condition of treatment with hrPTX3 and with the osteogenic cocktail compared to the untreated cells (*** *p* < 0.001) (Figure 5c).

## 3. Discussion

PTX3, a multifunctional glycoprotein, is known as a non-redundant component of the humoral arm of innate immunity and is, therefore, involved in triggering inflammatory mechanisms [21]. By interacting with specific ligands, such as C1q or microbial components, PTX3 can trigger complement system activation, opsonization of foreign antigens and phagocytosis by macrophages of late apoptotic neutrophils [22,23]. It is released by different cell types, such as fibroblasts, chondrocytes, vascular endothelial cells, adipocytes, smooth muscle cells, mesangial, epithelial and mesenchymal stromal cells, because of a variety of stimuli [24]. Through its complex quaternary structure, PTX3 is able to interact with a wide variety of molecules, including FGF2, thereby modulating the proliferation of endothelial and smooth muscle and the process of neo-angiogenesis [12,14]. It is also able to interact with extracellular matrix components such as hyaluronic acid and inter-alpha-trypsin inhibitor, thus playing an important role in extracellular matrix remodelling and female fertility [25,26].

In recent years, the role of PTX3 in bone homeostasis and its involvement in the mechanisms underlying the onset and progression of aging-related bone diseases have been investigated, but in this regard, conflicting data exist in the literature. In 2014, Lee and colleagues demonstrated that treatment with exogenous hrPTX3 was able to increase RANKL osteoblast production, inducing osteoclastogenesis [27]. In contrast, Kelava and colleagues demonstrated that Ptx3−/− mice were characterized by histomorphometric parameters indicative of bone loss, compared to wild-type (WT) mice, thus suggesting a positive role of PTX3 in bone metabolism [28]. In support of the results obtained by Kelava et al., we previously demonstrated that PTX3 is expressed in human osteoblasts. In this study, we demonstrated a significant reduction of the expression of PTX3 in the bone tissue of OP patients respect to both OA and CTRL groups. Furthermore, we showed that PTX3 inhibition significantly impaired both proliferation and mineralization activities of osteoblasts [2].

Based on these results, the aim of the present study was to further investigate the involvement of PTX3 in bone metabolism, analyzing its effect in comparison with classic osteoinduction treatment based on the use of β-glycerophosphate, dexamethasone and ascorbic acid.

First, we performed a histomorphometric analysis of the bone tissue to analyze conventional bone morphometric parameters, such as BV/TV, Tb.Th and Tb.S. In agreement with the literature, the measured data for all three parameters were indeed indicative of bone loss in OP patients compared to the CTRL group.

As expected, the immunohistochemical analysis showed that the bone tissue of OP patients was characterized by a significant increase of sclerostin expression. Conversely, a decrease in OPG expression was observed. These evidences are indicative of the higher rate of bone resorption generally observed in patients affected by osteoporosis. Immunohistochemical analysis of PTX3 expression showed that the protein was more highly expressed in CTRL subjects, both in hematopoietic bone marrow cells and in osteoblasts, than in OP patients. Specifically, in these subjects a very low number of PTX3 positive osteoblasts was detected. Further evidence that PTX3 is involved in the maintenance of bone homeostasis was derived from results obtained on primary osteoblast cultures. Treatment with hrPTX3 resulted in the acquisition of a mature osteoblast phenotype more clearly in the OP group than in the untreated condition, where a fibroblast-like appearance was evident. These differences were less pronounced in the CTRL group, whose cell cultures already had the basic appearance of mature osteoblasts. Noteworthily, compared to untreated and osteogenic cocktail conditions, hrPTX3 treatment also caused an increase in proliferation. The experiments carried out on primary osteoblast cultures allowed us to assess, in addition to the effects on cell proliferation and differentiation, the appearance of calcification-like structures following the two treatments. The amount of these structures was increased in OP cultures treated with hrPTX3 and osteogenic cocktail, while cultures isolated from bone tissue of healthy patients showed no significant difference between the three conditions. These results suggest a biological relationship between PTX3 and the ability of osteoblasts to form calcific nodules. In agreement with the literature, our results provide important evidence that PTX3 is a key factor involved in the maintenance of bone homeostasis. Treatment with hrPTX3 appears to restore the proliferative, differentiating, and mineralising capacity of osteoblasts isolated from OP patients, reestablishing the mature osteoblast phenotype already observed in cultures obtained from CTRL patients. The differential expression of PTX3, depending on the pathophysiological context, seems to be decisive on the action of this molecule. Indeed, while in an inflammatory context, high PTX3 expression is responsible for increased osteoclast-mediated bone resorption, our results suggest that the impairment of PTX3 expression can inhibit both osteoblasts proliferation and activity, thus inducing bone matrix depletion and the consequent risk of fractures.

## 4. Materials and Methods

All experimental procedures described in the present study were approved by the ethics committee of “Policlinico Tor Vergata” (approval reference number #85/12) and carried out according to The Code of Ethics of the World Medical Association (Declaration of Helsinki). Informed consent was obtained from all patients prior to surgery. Specimens were handled and carried out in accordance with the approved guidelines.

### 4.1. Patients

In this study, we enrolled five patients who underwent hip arthroplasty for fragility fracture (OP) and five patients who underwent hip arthroplasty for high energy fracture (CTRL) in the Orthopaedic Department of “Tor Vergata” University Hospital.

Patients with myopathies or other neuromuscular diseases or chronic administration of corticosteroid for autoimmune diseases (more than 1 month), alcohol abuse, cancer, diabetes and Hepatitis B Virus (HBV), Hepatitis C Virus (HCV), or Human Immunodeficiency Virus (HIV) infections were excluded from the study. Based on Dual-energy X-ray absorptiometry (DXA) results and radiographic assessment by the Kellgren–Lawrence scale [29], patients were divided into CTRL and OP. Clinical data of the patients are shown in Table 1.

### 4.2. Bone Mineral Density Evaluation

Lumbar spine (L1–L4) and femoral (neck and total) scans were performed through Dual-energy X-ray absorptiometry (DXA) with a Lunar DXA apparatus (GE Healthcare, Madison, WI, USA); bone mineral density (BMD) was measured according to manufacturer’s recommendations [30]. DXA measures BMD (in grams per square centimetre), with a coefficient of variation of 0.7%. For patients with fragility fractures, BMD was measured on the uninjured limb. For the other patients, measurements were performed on the non-dominant side, with the participants supine on an examination table with their limbs slightly abducted [31]. The DXA examination was performed 1 month after surgery and results were expressed as *T*-scores in Table 1.

### 4.3. Sampling

During surgery, bone head biopsies were collected and processed for histomorphometric and immunohistochemical analysis, and for setting up of primary osteoblast cultures.

### 4.4. Histology

Bone biopsies of the femoral head were fixed in 4% paraformaldehyde for 24 h and paraffin embedded without decalcification [32]. Un-decalcified tissues were cut by a tungsten carbide knife: 3 μm-thick sections were stained using haematoxylin and eosin (H&E) (Bio-Optica, Milan, Italy) to perform histomorphometric analysis and 3 μm-thick sections were processed for immunohistochemical analysis.

### 4.5. Histomorphometric Analysis

Ten microscopic images, randomly selected, were evaluated for each biopsy sample. Images were acquired at 2x magnification using a Nikon upright microscope ECLIPSE Ci-S/(Nikon Corporation, Tokyo, Japan) connected to a Nikon digital camera. Image analysis was performed using NIS-Elements software (5.30.01; Laboratory Imaging, Prague, Czech Republic), according to the manufacturer instructions [33]. The parameters BV/TV, Tb.Th, and Tb.Sp were evaluated according to Dempster et al. [34].

### 4.6. Immunohistochemistry

Sclerostin, OPG and PTX3 expression were assessed in bone biopsies by immunohistochemistry analysis. 3 μm-thick sections were pretreated with EDTA citrate pH 7.8 for 30 min at 95 °C and then incubated with rabbit polyclonal anti-sclerostin antibody for 60 min (1 μg/mL, clone NA, AbCam, Cambridge, United Kingdom), and mouse monoclonal anti-OPG for 60 min (1 μg/mL, clone 98A1071, Novus Biologicals, Littleton, CO, USA). For PTX3 expression evaluation, sections were pretreated with citrate pH 6 for 30 min at 95 °C and then incubated with rat monoclonal anti-PTX3 for 120 min (2 μg/mL, clone MNB1, AbCam). Washing was performed with phosphate-buffered saline (PBS)/Tween20 pH 7.6 (UCS Diagnostic, Rome, Italy); reactions were revealed by horseradish peroxidase (HRP)-3,3′ diaminobenzidine (DAB) Detection Kit (UCS Diagnostic). To assess the background of immunostaining, we included a negative control for each reaction by incubating the sections with secondary antibodies (HRP) and a detection system (DAB).

Immunohistochemical positivity was assessed on digital images acquired with NIS-Elements software using a semi-quantitative approach, scoring from 0 to 3 based on the number of positive osteocytes out of the total analysed for sclerostin and positive osteoblasts for OPG and PTX3. For each sample, 50 osteoblasts or osteocytes were assessed and scored as shown in Table 2.

### 4.7. Human Osteoblast Primary Cell Cultures

To obtain primary cultures of osteoblasts, trabecular bone fragments were repeatedly washed in PBS. Then, bone fragments were briefly incubated at 37 °C with 1 mg/mL Trypsin from porcine pancreas ≥60 U/mg (SERVA Electrophoresis GmbH Heidelberg, DE) diluted in Dulbecco’s Phosphate-Buffered Saline (DPBS). After washing, bone fragments underwent to repeated digestions with 2.5 mg/mL Collagenase NB 4G Proved grade ≥0.18 U/mg (SERVA Electrophoresis GmbH, Heidelberg, Germany) diluted in DPBS with calcium and magnesium. Supernatant were collected and centrifuged at 310 RCF for 5 min. Cell pellets were resuspended in Dulbecco’s modified Eagle medium (DMEM) with 15% fetal bovine serum (FBS), seeded into a 24-wells plate, and incubated at 37 °C 5% CO_2_ until reaching confluence (about 4 weeks). Medium was changed twice a week.

### 4.8. Immunostaining of Primary Cell Cultures

Expression of PTX3 was evaluated by immunofluorescence in CTRL and OP primary osteoblast cultures. After fixation in PFA 4% for 30 min, cell cultures were pretreated with Ethylenediaminetetraacetic acid (EDTA) citrate pH 7.8 for 20 min at 95 °C and incubated with rat monoclonal anti-PTX3 antibody for 60 min (2 μg/mL, clone MNB1, AbCam). Reaction with anti-PTX3 was revealed by using UltraTek Anti-Polyvalent Biotinylated antibody (ScyTek Laboratories, Logan, UT, USA). Washing was performed with PBS/Tween 20 pH 7.6 (UCS Diagnostic, Rome, Italy). Finally, cells were counteracted with 4’,6-diamidino-2-phenylindole (DAPI) counterstain (Kreatech Biotechnology B.V., Amsterdam, The Netherlands).

### 4.9. Osteoblast Primary Cultures Conditioned with hrPTX3 and Osteogenic Cocktail

Cells from the first or second passage were seeded into a 24-wells plate at a density of 10 × 103 cells/well. The following conditions were verified: (a) untreated primary cultures (72 h); (b) primary cultures treated with hrPTX3 (20 ng/mL, 72 h); (c) primary cultures treated with osteogenic cocktail (DMEM F12, FBS 10%, Penicillin/Streptomycin (P/S), L-Glutamine (L-GLU)/stable, β-glycerophosphate: final concentration 10 μM, Ascorbic acid: final concentration 50 μg/mL, Dexamethasone: final concentration 100 nM, 72 h). Cell’s differentiation and proliferation were studied by both Toluidine blue staining and SEM-EDX microanalysis.

### 4.10. Statistical Analysis

All statistical analyses were performed using GraphPad Prism 8 Software (Prism 8.0.1, La Jolla, CA, USA). Clinical data were analyzed by the Mann–Whitney test. Immunohistochemical and histomorphometric parameters were analyzed by one-way analysis of variance (ANOVA), Tukey’s test and Dunnett’s multiple comparison test and were considered significantly different if *p* < 0.05.

## 5. Conclusions

Overall, this study provides additional information on potential mechanisms underlying the onset of bone fragility in subjects affected by osteoporosis, confirming the role played by PTX3 in bone metabolism. In addition, the results reported here can lay the foundation for further studies focused on the possible use of PTX3 as biomarkers and treatment option for OP patients.

## Figures and Tables

**Table 1:** Clinical data of CTRL and OP patients.

Parameters	CTRL	OP	*t*-Test (Mann–Whitney Test)
Age (years)	49.1 ± 1.3	78.5 ± 2.1	*** *p* < 0.001
*T*-score (L1–L4)	1.1 ± 0.03	−2.5 ± 0.5	** *p* < 0.01
*T*-score (femoral neck)	1.5 ± 0.2	−2.7 ± 0.14	** *p* < 0.01
*T*-score (total femur)	1.3 ± 0.3	−2.1 ± 0.3	** *p* < 0.01
Phosphorous (mg/dl)	3.8 ± 0.4	2.6 ± 0.2	* *p* < 0.05
Calcium (mg/dl)	6.99 ± 0.12	9.5 ± 0.2	* *p* < 0.05

CTRL: control patients; OP: osteoporotic patients.

**Table 2:** Scoring system for immunohistochemical analysis.

SCORE	0	1	2	3
**Positive Cells**	≤2	3 ≤ x ≤ 12	13 ≤ x ≤ 22	≥23

## Data Availability

The data presented in this study are available on request from the corresponding author.
